# Explore potential immune-related targets of leeches in the treatment of type 2 diabetes based on network pharmacology and machine learning

**DOI:** 10.3389/fgene.2025.1554622

**Published:** 2025-04-14

**Authors:** Tairan Hu, Zhaohui Fang

**Affiliations:** ^1^ Anhui University of Traditional Chinese Medicine, Hefei, China; ^2^ Department of Cardioiogy, First Hospital Affiliated to Anhui University of Traditional Chinese Medicine, Branch of National Clinical Research Center for Chinese Medicine Cardiology, Institution of Cardiovascular Disease, Anhui Academy of Chinese Medicine Sciences, Hefei, China

**Keywords:** leech, type 2 diabetes, network pharmacology, immune infiltrate, machine learning

## Abstract

**Introduction:**

Type 2 diabetes mellitus (T2DM) is a chronic metabolic disorder that poses a significant global health burden due to its profound effects on systemic physiological homeostasis. Without timely intervention, the disease can progress insidiously, leading to multisystem complications such as cardiovascular, renal, and neuropathic pathologies. Consequently, pharmacological intervention becomes crucial in managing the condition. Leeches have been traditionally used in Chinese medicine for their potential to inhibit the progression of T2DM and its associated complications; however, the specific mechanisms underlying their action and target pathways remain poorly understood. The objective of this study was to predict potential therapeutic targets of leeches in the treatment of T2DM.

**Methods:**

We collected active components and targets associated with leeches from four online databases, while disease-related targets were sourced from the GeneCards and OMIM databases. Following this, we performed Gene Ontology (GO) analysis and Kyoto Encyclopedia of Genes and Genomes (KEGG) enrichment analysis. Gene expression data were obtained from the GSE184050 dataset. Important immune cell types were identified through immunoinfiltration analysis in conjunction with single sample enrichment analysis (ssGSEA). Additionally, weighted co-expression network analysis (WGCNA) was utilized to identify significantly associated genes. Finally, we employed LASSO regression, SVM-RFE, XGBoost, and random forest algorithms to further predict potential targets, followed by validation through molecular docking.

**Results:**

Leeches may influence cellular immunity by modulating immune receptor activity, particularly through the activation of RGS10, CAPS2, and OPA1, thereby impacting the pathology of Type 2 Diabetes Mellitus (T2DM).

**Discussion:**

However, it is important to note that our results lack experimental validation; therefore, further research is warranted to substantiate these findings.

## 1 Introduction

Type 2 diabetes is a chronic disease that seriously threatens human health (Kautzky-Willer et al., 2016). Current studies suggests that the pathogenesis of type 2 diabetes mellitus is primarily attributed to both insulin resistance and impaired systemic capacity to metabolize surplus glucose (Lee et al., 2022). Diminished cellular glucose oxidation (DeFronzo, 2004) and dysregulated mitochondrial bioenergetics (Rovira-Llopis et al., 2017) constitute significant contributors to the pathogenesis of type 2 diabetes mellitus (T2DM). Pharmacotherapy remains the primary therapeutic approach for type 2 diabetes mellitus (T2DM); however, adverse drug reactions persist as a significant clinical challenge affecting a substantial patient population.

Leech-derived bioactive constituents demonstrate targeted anti-hyperglycemic efficacy, while concomitant administration with exogenous insulin achieves synergistic glycemic regulation, thereby overcoming the therapeutic constraints inherent to conventional monotherapeutic approaches targeting isolated pathways (Mohammed et al., 2013). Concurrently, experimental studies have revealed that leech therapy ameliorates streptozotocin (STZ)-induced nephrotic microangiopathy in diabetic rats by suppressing endothelial cell migration and angiogenesis (Pang et al., 2020). Hirudin ameliorates kidney injury in diabetic nephropathy by suppressing GSDMD-mediated pyroptosis, thereby reducing the release of renal inflammatory factors (e.g., IL-1β and IL-18) (Han et al., 2023). The bioactive components of leeches ameliorate diabetic nephropathy and preserve renal function through anticoagulant, antifibrotic, antithrombotic, and anti-inflammatory mechanisms (Tian et al., 2024). These results suggest that leeches may play a role in the treatment of T2DM, however, the specific mechanisms require further study.

Network pharmacology is an effective method to explore the underlying mechanisms of drugs and diseases, and the mechanism of action and potential targets of leech therapy for T2DM are explored in combination with immunoinfiltration analysis, and verified by molecular docking.

## 2 Materials and methods

### 2.1 Network pharmacological analysis

To investigate the mechanisms underlying the therapeutic effects of leech therapy in diabetes, we performed a network pharmacology-based analysis. First, the active ingredients of leeches were identified using the Herb database (http://herb.ac.cn/) (Fang et al., 2021), with data retrieval performed through the “Related Ingredients” section. The SMILES notation of the ingredients, which represents their structural formulas, was obtained from the PubChem database. These notations were subsequently utilized for target prediction analyses. Target data through SwissTargetPredictio (https://www.swisstargetprediction.ch/) (Daina et al., 2019), CTD (https://ctdbase.org/) (Davis et al., 2023), SuperPred (Database (http://prediction.charite.de/) (Gallo et al., 2022), disease targets from Genecards (www.genecards.org), OMIM (https://omim.org/) (Stelzer et al., 2016) database access, Keyword “Type 2 diabetes” was set, and disease targets with Relevance score > 10 were screened. The leech-active component-target network was generated using Cytoscape (version 3.8.2). Node degree centrality was calculated to quantify connection patterns, with higher values indicating greater nodal influence within the network architecture. Using the “cytohuub plugin” in Cytoscape software, select “Degree” to obtain the main active ingredients of leeches. Finally, microscopic letter platform (http://www.bioinformatics.com.cn/) to further optimize the “leech - active ingredient - targets” network diagram. R software “clusterProfiler” “pathview” package for enrichment analysis.

### 2.2 Obtain GEO datasets and GSEA analysis for T2DM

The GSE184050 dataset was retrieved from the Gene Expression Omnibus (GEO) database (http://www.ncbi.nlm.nih.govgeo) (Barrett et al., 2013). This dataset comprises 116 whole blood transcriptome samples, including fifty patients with type 2 diabetes and sixty-six healthy controls. The Series Matrix File and SOFT formatted files were obtained from the database to facilitate data normalization processes. Differential expression analysis was performed using GEO2R, with the disease and control groups defined to generate differentially expressed genes (DEGs). These DEGs were subsequently processed in R (version 4.2.1) for downstream analyses. Finally, differential analysis was conducted using the limma package (Ritchie et al., 2015) in R software, with significance thresholds defined as p < 0.05 and absolute fold change (|FC|) > 1. Finally, a differential expression heatmap was generated from the expression profile dataset. Gene Set Enrichment Analysis (GSEA) (Canzler and Hackermüller, 2020) was subsequently conducted to analyze enriched gene sets. Through GSEA analysis, the gene ontology (GO) and associated signaling pathway (KEGG) of DEGs were determined.

### 2.3 Single sample enrichment analysis (ssGSEA)

Immune-related gene set enrichment in the study samples was assessed using single-sample gene set enrichment analysis (ssGSEA) (Jin et al., 2021), a modified version of traditional GSEA specifically designed for evaluating predefined gene set enrichment at the individual sample level. This method enables independent evaluation of immune pathway and related process activity within individual samples, eliminating the need for comparative data from other specimens. Transcriptome data was annotated by GRCh38 genome annotation file, and mRNA was extracted and normalized into tpm for subsequent analysis (Yi et al., 2020).

### 2.4 Weighted gene coexpression network analysis (WGCNA)

WGCNA is designed to identify co-expression patterns of gene modules associated with specific biological processes, diseases, or other phenotypes (Xu et al., 2023; Feng et al., 2022). In this study, we constructed a gene co-expression network using all expressed genes (DEGs) and activated CD4 cell scores, activated dendritic cell scores, central memory CD4 T cell scores, and type 1 T helper cell scores in the dataset. Firstly, the power scatter plot was established and the optimal soft threshold (β = 12) was selected to obtain the similarity matrix between the genes. Then, hierarchical clustering of genes was performed, and dynamic module identification and clipping were performed with the minimum module number of 80. The resulting gene modules were visualized by co-expression patterns, and similar modules were combined. Correlations between activated DC scores and each module were assessed by correlation tests, and heat maps were drawn to show the correlation results.

### 2.5 Machine learning analysis of intersection targets

Immune-related targets for leeches to treat diabetes were screened by machine learning algorithms. First, the intersection targets of leeches and WGCNA core modules were selected as objects, and the targets were further screened by LASSO regression (Hohberg and Groll, 2024), SVM-REF (Yang et al., 2024), xgboost algorithm (Hou et al., 2020) and random forest algorithm (Hu and Szymczak, 2023). LASSO regression is a feature screening algorithm. The intersection targets are set as high data, and the ten-fold crossover algorithm is used to avoid overfitting and maintain the accuracy of the algorithm. SVM-REF algorithm is a classification algorithm, which reduces the error through classification and screening method. Xgboost algorithm and random forest algorithm can process data more efficiently, and process variables through cross-analysis method to realize high-latitude data sorting.

### 2.6 Molecular docking

The macromolecular structures were downloaded from the PDB website (Prestegard, 2021), specifically for RGS10, CAPS2, and OPA1, with PDB numbers 2DLR, Q86UW7, and 6JT, respectively. The small molecule structure compositions were sourced from PubChem (https://pubchem.ncbi.nlm.nih.gov/) (Kim, 2016), while the CAPS2 molecular structure was obtained from AlphaFold (https://alphafold.ebi.ac.uk). AutoDockTools-1.5.7 was used to process both small and large molecules. This involved “dehydrating and hydrogenating,” setting the docking box, and calculating the binding energy based on the number of hydrogen bonds and interactions between the small and large molecules. For visualization, we utilized PyMOL 3.1, presenting the final results with both overall and partial views of the docking.

### 2.7 Statistical analysis

All statistical analyses were conducted using R software (version 4.3.1), with a p-value of less than 0.05 deemed statistically significant. For network pharmacology investigations, Cytoscape (version 3.8.2) and its plugins were utilized to construct and analyze networks. The topological assessment of the “drug-component-target” network was primarily based on node degree centrality. Protein-protein interaction (PPI) network clusters were identified using Cytoscape’s MCODE plugin with the following parameters: degree cut-off = 2, node score cut-off = 0.2, K-core = 2, and maximum depth = 100. Core targets were subsequently determined through node degree ranking. Molecular docking simulations were performed with an energy range of 5 kcal/mol and exhaustiveness set to 400.

## 3 Results

### 3.1 Network pharmacological analysis

**FIGURE 1:**
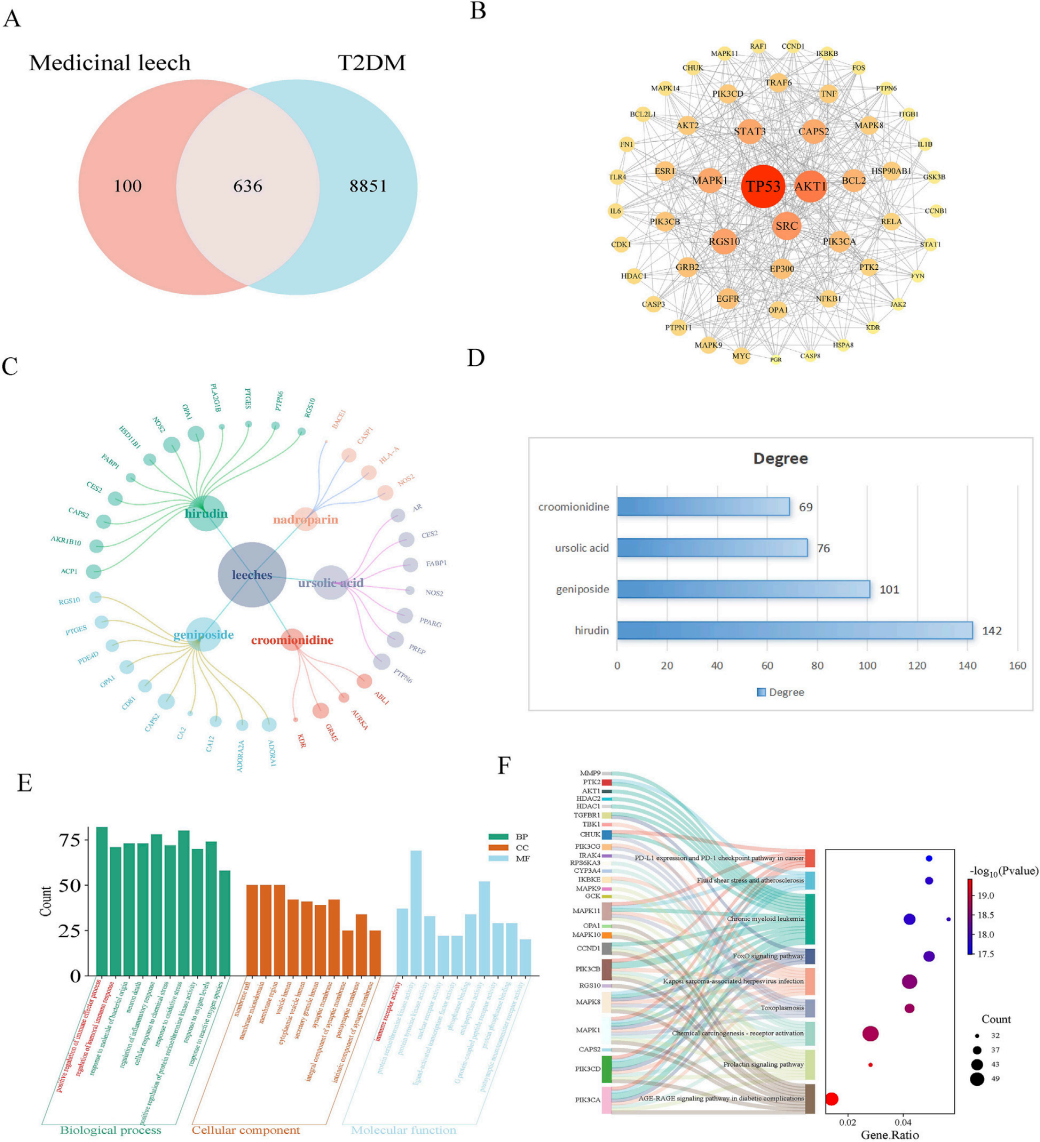
**(A)** Venn diagram of leeches and T2DM targets; **(B)** PPI maps of intersection targets. In the PPI picture of the intersection target, the size of the circle represents the correlation, and the color represents the P-value. The smaller the P-value, the darker the color. **(C)** The network diagram for “Leech - active ingredient - target” illustrates the relationships between different components. In this diagram, the size of each circle indicates the importance of the composition, with larger circles signifying a stronger correlation. The primary active components of leeches include hirudin, geniposide, ursolic acid, and croomionidine, which play key roles in their medicinal properties. **(D)** Table of degree values of main active ingredients of leech. **(E)** The GO function analysis histogram visually presents the different categories of gene functions. In this representation, Biological Processes (BP) are marked in dark cyan, Cellular Components (CC) in sienna, and Molecular Functions (MF) in steel blue. The analysis indicates that leeches are significantly involved in the positive regulation of immune processes, humoral immune response, and inflammatory response under Biological Processes. Furthermore, they play a role in Molecular Functions related to immunoreceptor activity and protein serine/threonine kinase activity. **(F)** Sankey plot by KEGG enrichment analysis. Shows the link between signaling pathways and genes.

A total of 19 active components from leeches were obtained through the HERB database. Subsequently, 461 component targets were identified using SWI, while 96 drug targets were retrieved from the CTD, and 258 drug targets were sourced from SuperPred. After removing duplicates, a total of 736 unique drug targets were identified. A total of 9,487 disease targets were identified through screenings from GeneCards and OMIM. The results revealed that there are 636 common targets associated with both leeches and diabetes mellitus (Figure 1A). Protein interaction analysis was also performed (Figure 1B). The component-target network illustrated the relationship between the effective components and targets of leeches (Figure 1C). The results indicate that the main active ingredient of leeches is hirudin, geniposide, ursolic acid, croomionidine (Figure 1D). The results of the GO enrichment analysis for biological processes indicated that leeches are involved in the positive regulation of immune response, humoral immune response, oxidative stress response, and inflammatory response. The molecular function analysis revealed that Hirudo is involved in immune receptor activity, protein serine/threonine kinase activity, and protein tyrosine kinase activity (Figure 1E); KEGG results showed involvement in AGE-RAGE signaling pathway and chemical carcinogenic-receptor activation signaling pathway (Figure 1F).

### 3.2 GSEA analysis of differential genes

**FIGURE 2:**
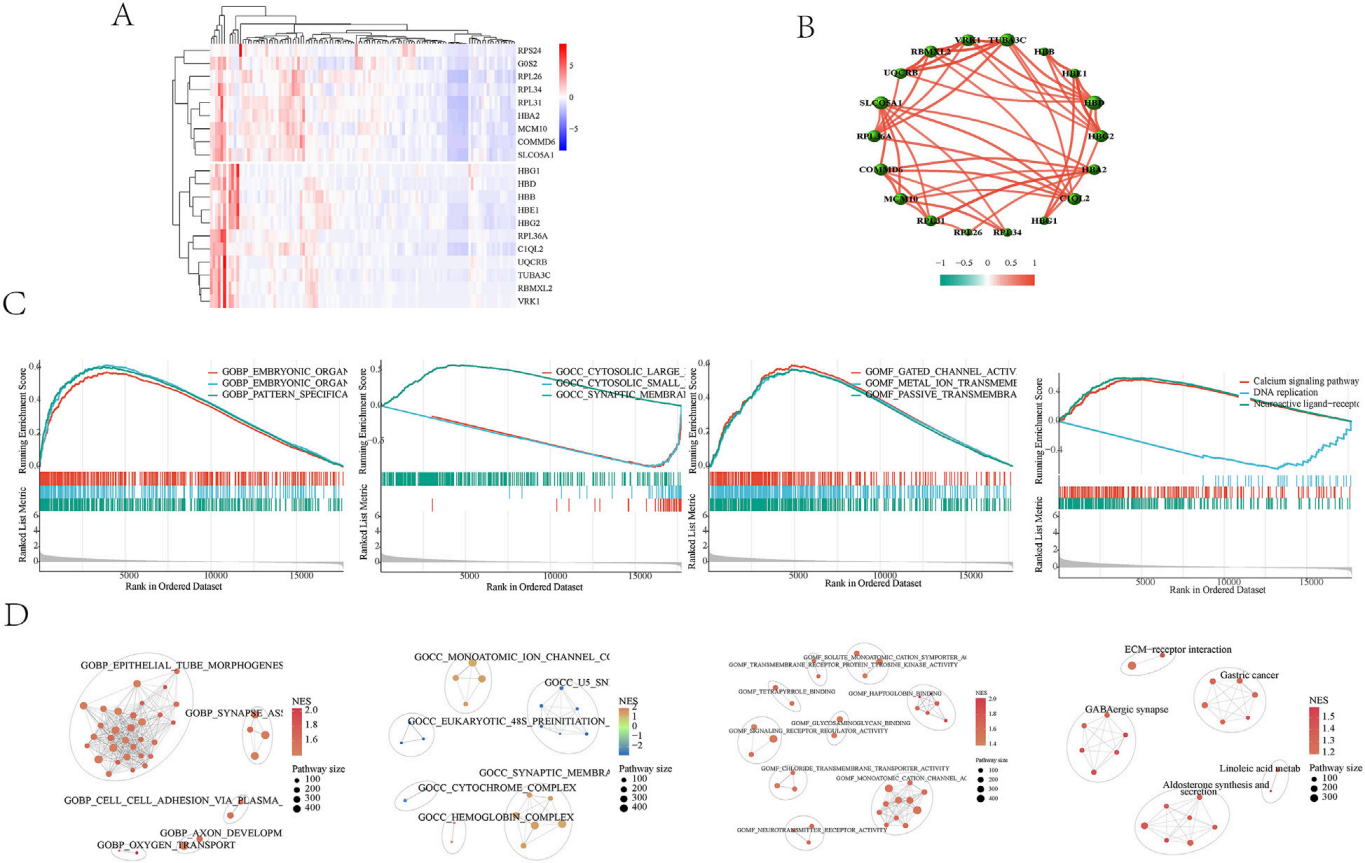
**(A)** Heat map of differential analysis. The top 20 genes in the differential analysis were shown. **(B)** Correlations among the top 20 differentially expressed genes were established. **(C)** GSEA Enrichment Analysis Diagram. The results indicated that bioengineering primarily influences organic morphogenesis, organ development, and the skeletal system. The KEGG pathway analysis highlighted that the main focus is on signaling pathways, including neural active ligand-receptor interactions, calcium signaling, and DNA replication. The enrichment results were categorized into different gene clusters based on gene classification. **(D)** GSEA enriched cell cluster. GSEA enriched cell clusters were categorized into different gene clusters based on gene classification.

Whole blood transcriptome data were obtained from the GSE184050 dataset, which included 116 patients—50 with type 2 diabetes and 66 healthy controls. By standardizing the sample data and using gene IDs instead of probe IDs, expression profiles for both the normal and disease groups were constructed. A total of 17,732 gene data were collected, resulting in the identification of 3,939 differentially expressed genes that were upregulated. The heat map shows the significantly different genes (Figure 2A). Correlations among the top 20 differentially expressed genes were established (Figure 2B). GSEA analyzed the Gene Ontology (GO) and Kyoto Encyclopedia of Genes and Genomes (KEGG) related to disease genes. The results indicated that bioengineering primarily influences organic morphogenesis, organ development, and the skeletal system (Figure 2C). In terms of molecular functions, it is involved in transmembrane transport and organ development. The KEGG pathway analysis highlighted that the main focus is on signaling pathways, including neural active ligand-receptor interactions, calcium signaling, and DNA replication. The enrichment results were categorized into different gene clusters based on gene classification (Figure 2D). These findings suggest that maintaining the stability of the body’s internal environment and the functionality of cell receptors play significant roles in the development of Type 2 Diabetes Mellitus (T2DM).

### 3.3 Immunoinfiltration and single sample enrichment analysis (ssGSEA)

**FIGURE 3:**
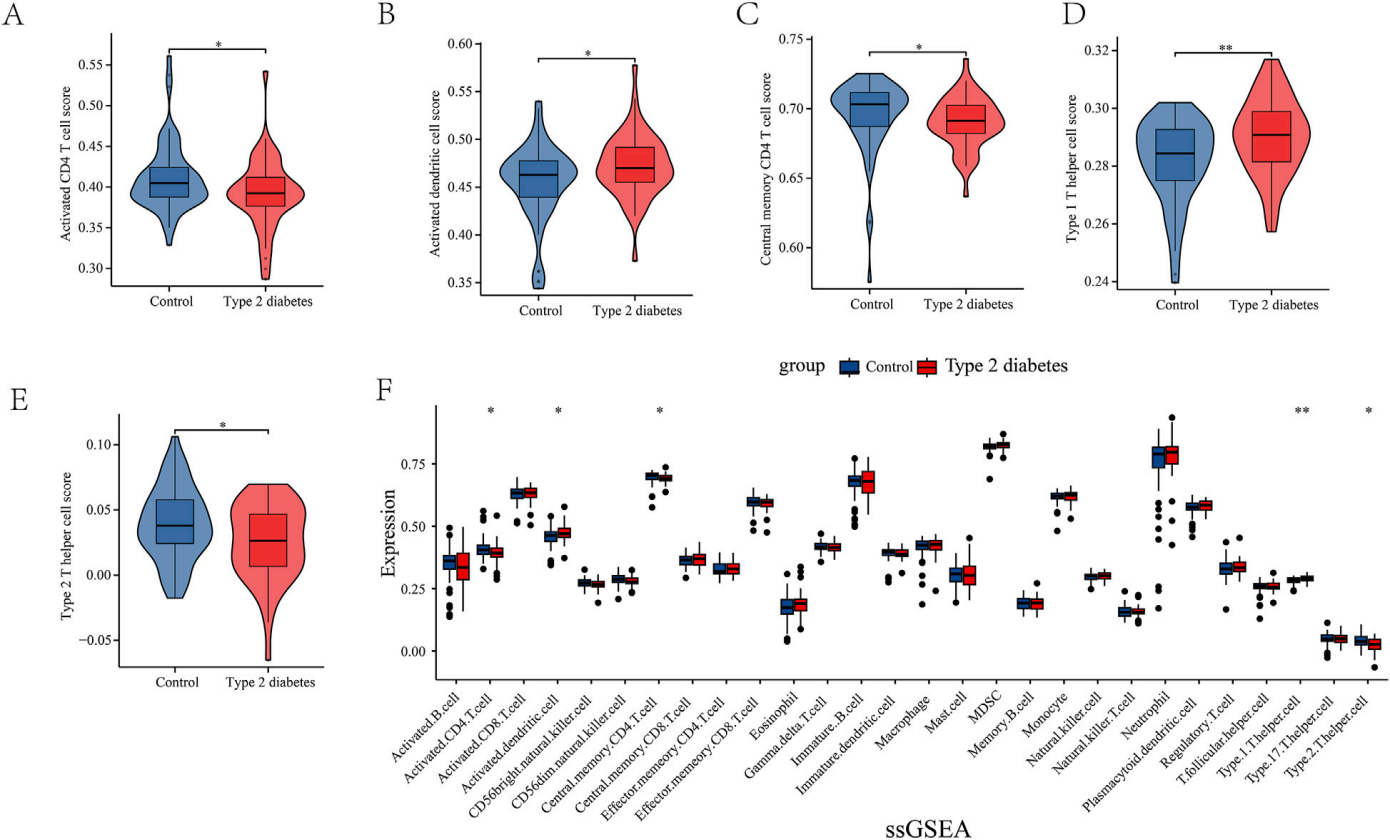
**(A–F)** Single Sample enrichment analysis (ssGSEA) of important immune cells. They were activated CD4 cells, activated dendritic cells, central memory CD4 T cells, type 1 T helper cells, and type 2 T helper cells.

Through network pharmacology results, we found that leeches may be involved in the regulation of type 2 diabetes through immunomodulatory regulation of relevant immune cells and receptors. In order to obtain immune-related targets, we performed immunoinfiltration analysis. Gene sets for 28 immune cells, encompassing a total of 782 genes, were downloaded from the TISIDB database. The results are presented in (Figures 3A–E). We also conducted ssGSEA analysis, which revealed that several key immune cells were involved. Activated CD4 cells and central memory CD4 T cells were highly expressed in the normal group, while activated dendritic cells, type 1 T helper cells, and type 2 T helper cells showed higher expression levels in the T2DM group (Figure 3F).

### 3.4 Analysis of weighted gene co-expression network

**FIGURE 4:**
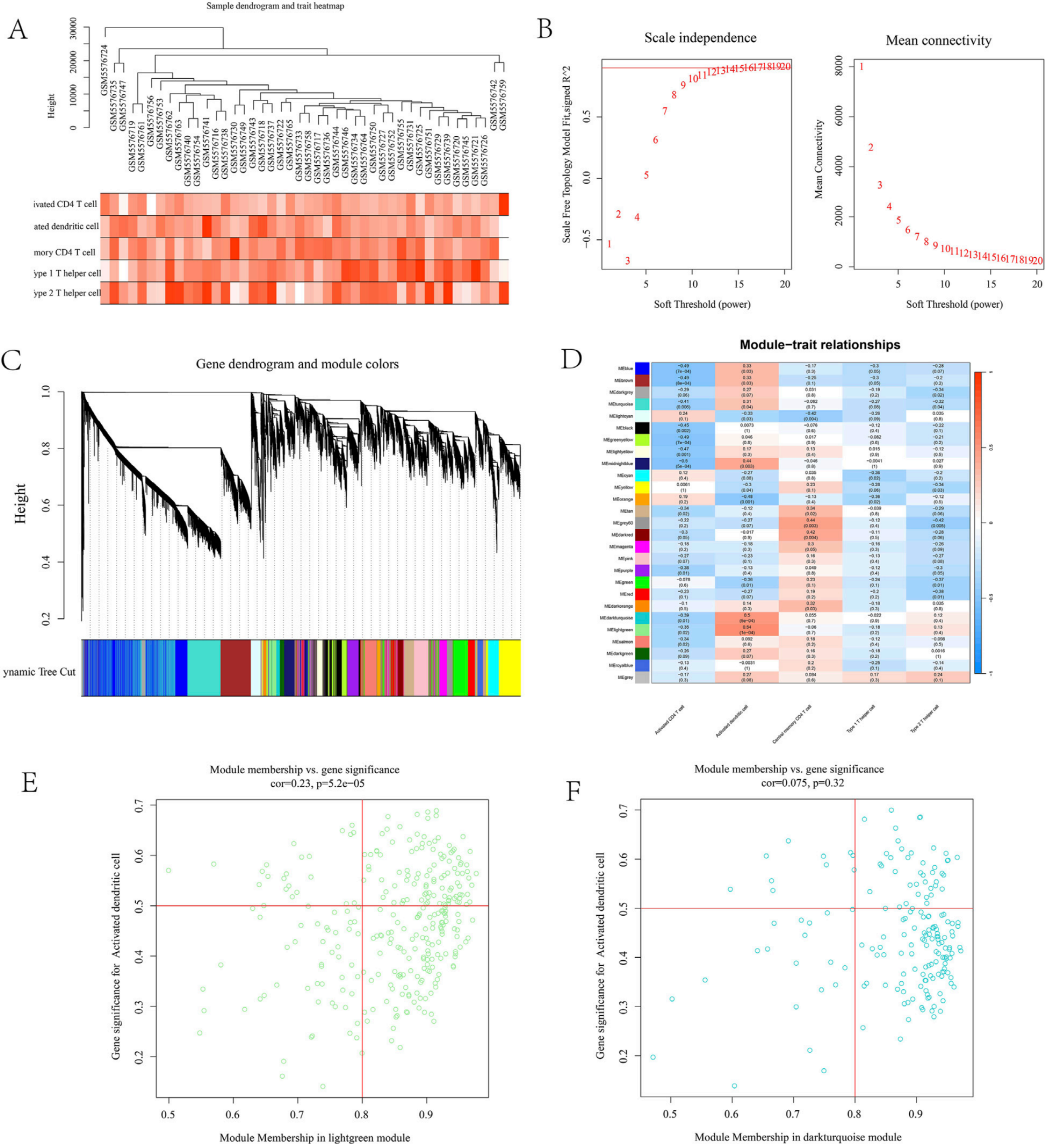
**(A)** Expression differences in sample data; **(B)** represents connectivity graph; **(C)** sample represents cluster graph; **(D)** module correlation heat map. The darker the color, the stronger the correlation. MEdarkturquoise module (5e-04) and MElightgreen module (1e-04) had the strongest correlation, both belonging to activated dendritic cells; **(E)** MM and GS correlation coefficient maps. MEdarkturquoise module are positively correlated (cor = 0.075, p = 0.32); **(F)** MM and GS coefficients of MElightgreen module were positively correlated (cor = 0.23, p = 5.2e-05).

To further identify immune-related targets for T2DM treatment in leeches, we conducted WGCNA analysis. Gene co-expression networks were constructed for activated CD4 cells, activated dendritic cells, central memory CD4 T cells, type 1 T helper cells, and type 2 T helper cells using the GSE184050 dataset. Assessment of outliers No significant outliers were detected in the data (Figure 4A). A soft threshold power assessment of 10, with a scale-free index of 0.9, indicates that the connectivity within the network is reasonable (Figure 4B). The correlation trend and correlation heatmap among different samples were generated, and a multi-level modular clustering tree was created using a weighted co-representation network (Figure 4C). The final results indicated that all samples were categorized into a normal group and a disease group, which were further divided into 26 distinct modules. The correlation between these modules was represented by P-values (Figure 4D). The correlation coefficients between phenotypes and modules were calculated. The results revealed that the MEdarkturquoise module (cor = 0.5, p = 5e-04) and the MElightgreen module (cor = 0.54, p = 1e-04) displayed the most significant correlations with Type 2 Diabetes Mellitus (T2DM). Module membership (MM) and gene significance (GS) reflect the correlation of genes within a particular module. The scatter plot shows that the MM and GS coefficients of MEdarkturquoise module are positively correlated (cor = 0.075, p = 0.32), MM and GS coefficients of MElightgreen module were positively correlated (cor = 0.23, p = 5.2e-05). The MEdarkturquoise module and the MElightgreen module may be critical modules that play the most significant roles in Type 2 Diabetes Mellitus (T2DM), and they are also associated with activated dendritic cells. (Figures 4E,F).

### 3.5 Machine learning

**FIGURE 5:**
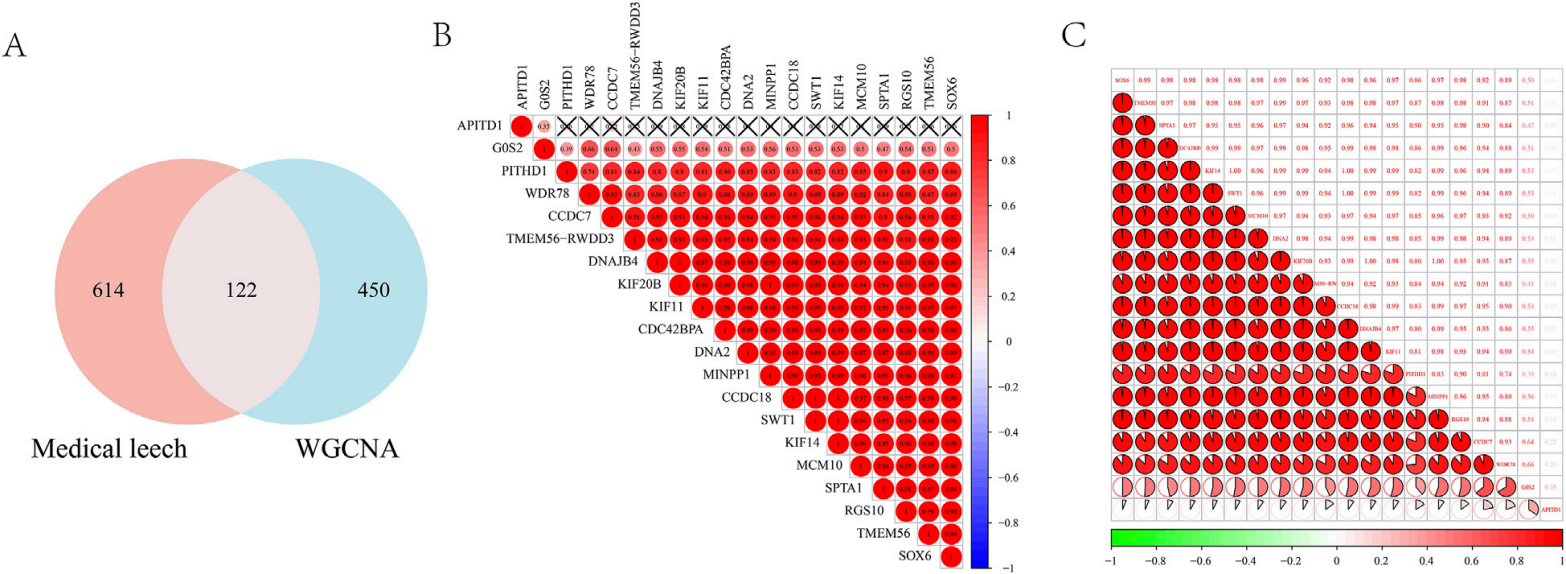
**(A)** Wynn diagram of drug targets and WGCNA core module targets; Correlation heat map of the top 20 intersection targets in **(B, C)**.

**FIGURE 6:**
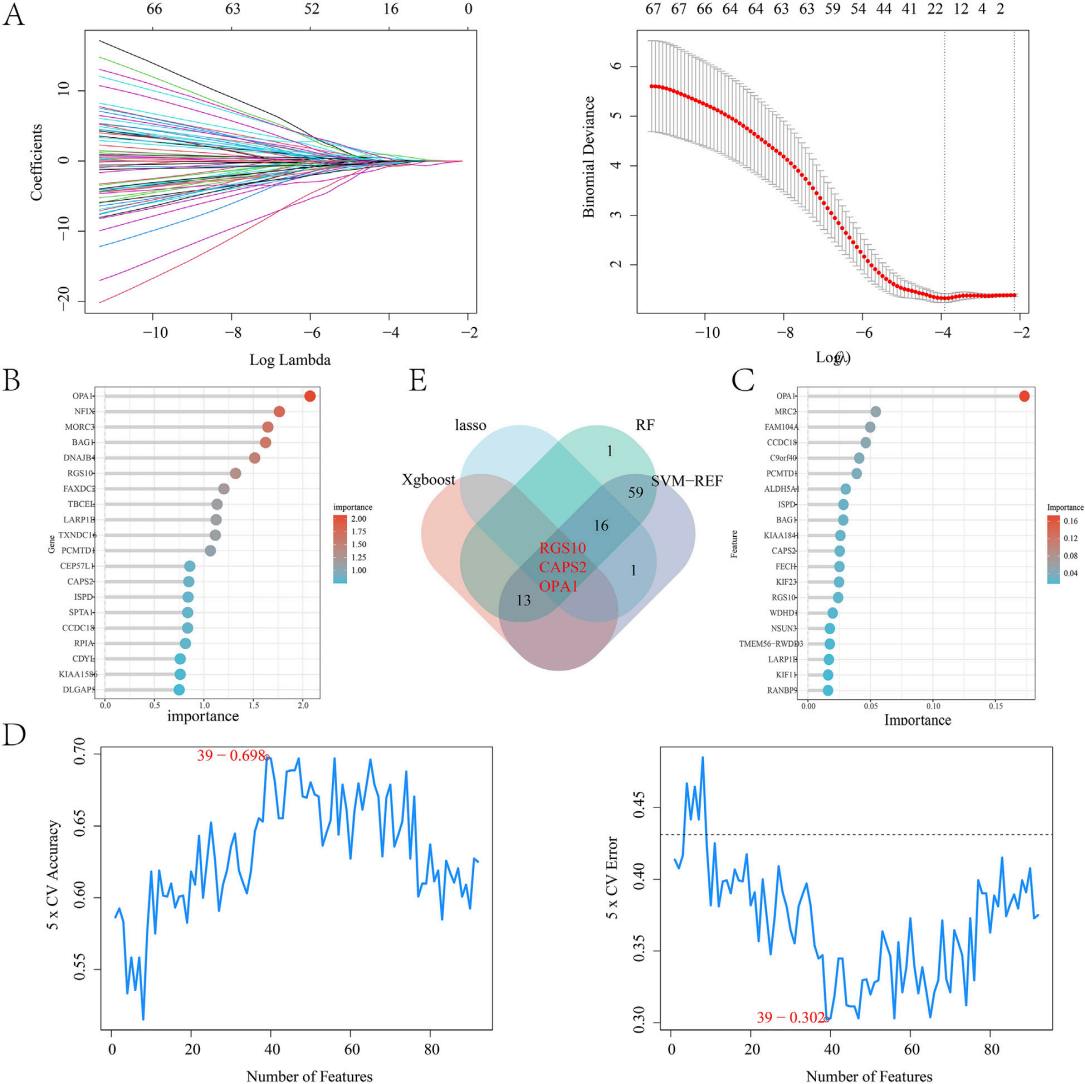
**(A)** LASSO regression model; **(B)** Random forest algorithm model; **(C)** Xgboost algorithm model; **(D)** SVM-REF algorithm model; **(E)** Wayne diagram of intersection targets obtained by four algorithms.

By intersecting the leech targets with those analyzed through WGCNA, a total of 122 overlapping targets were identified, which served as the focus for machine learning (Figure 5A). The correlation among the top 20 targets is illustrated in a heatmap (Figures 5B,C). In the LASSO regression analysis, we performed 10-fold cross-validation (10-fold CV) using cve. glmnet to optimize the hyperparameter λ, and selected the optimal λ value based on binomial deviance: λ_min = 0.02197 (corresponding to 13 features). λ_1se = 0.11727 (all features are compressed to 0, the regularization intensity is high). And 13 key genes were selected (Figure 6A). During the training process of the random forest model, we determined the optimal mtry value using tuneRF and selected the corresponding ntree that yielded the minimum out-of-bag (OOB) error for hyperparameter optimization. Finally, we selected the top 20 key genes based on the feature importance ranking (Figure 6B). During the XGBoost training, we used eval_metric = “error” to monitor model errors and selected the best model based on the final training errors. To optimize model performance, we adjusted hyperparameters such as max_depth, eta, and gamma through Grid Search (Figure 6C). SVM-RFE employed 10-fold cross-validation to assess model performance and ensure the generalization capability of feature selection. This method divided the data into training and test sets, resulting in the identification of 39 variables to build the model (Figure 6D). Ultimately, we identified three common targets: RGS10, CAPS2, and OPA1 (Figure 6E).

### 3.6 Molecular docking

**FIGURE 7:**
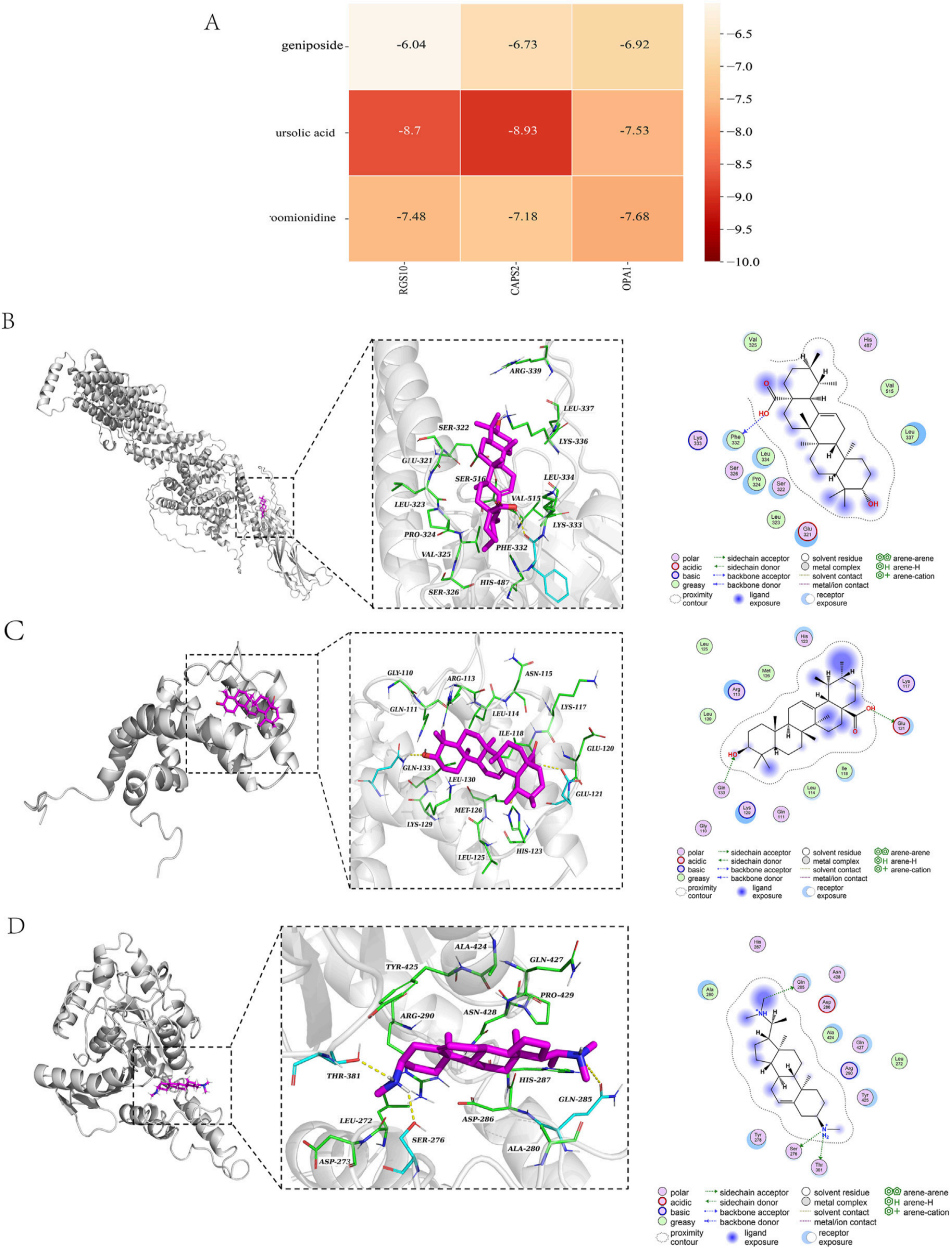
**(A)** Table of docking scores; **(B)** CAPS2-ursolic acid molecular docking diagram; **(C)** RGS10-ursolic acid molecular docking diagram; **(D)** OPA1-croomionidine molecular docking diagram.

Due to the large molecular weight of hirudin, the error in the molecular docking process is significantly affected; therefore, the hirudin component was excluded from the docking analysis. To better validate our results, geniposide, ursolic acid, and croomionidine were selected along with CAPS2, RGS10, and OPA1 as the top three components for docking analysis, respectively. The binding heat energy was less than -5 kcal/mol, indicating good bonding results. The results indicated that the binding affinity of CAPS2-ursolic acid is −8.9 kcal/mol, with a docking score of −8.93 kcal/mol. Additionally, the binding affinity of OPA1-croomionidine is −7.37 kcal/mol. The docking score is −7.68 kcal/mol, while the minimum binding affinity of RGS10-ursolic acid is −8.69 kcal/mol, and the docking score is −8.7 kcal/mol. The binding affinities and docking scores are largely consistent, indicating a strong binding strength between the ligand and the receptor (Figures 7B–D). All docking scores are displayed in Figure 7A.

## 4 Discussion

Type 2 diabetes mellitus (T2DM) constitutes a major global health burden, with its associated complications significantly compromising patient quality of life. Current clinical management primarily relies on pharmacological interventions; however, therapeutic outcomes demonstrate significant interindividual variability (Kautzky-Willer et al., 2016). To improve disease control, optimize treatment efficacy, and mitigate complication risks, the discovery of novel therapeutic agents and mechanisms remains critically imperative. Emerging evidence demonstrates the therapeutic potential of Traditional Chinese Medicine (TCM) in T2DM management, particularly in ameliorating diabetic nephropathy (Cao et al., 2025). Furthermore, multimodal therapeutic strategies for T2DM include metabolic regulation through reduction of serum triglyceride and cholesterol levels (Tong et al., 2018), as well as pharmacological modulation of α-glucosidase activity (Zhou et al., 2023).

Hirudo (leech), an animal-derived traditional Chinese medicine, has been historically employed for its therapeutic properties in activating blood circulation and resolving stasis. Empirical studies have validated its antidiabetic efficacy, particularly through its principal bioactive component - hirudin. This naturally occurring thrombin inhibitor (Greinacher and Lubenow, 2001), identified as a core bioactive constituent in our network pharmacological analysis, exhibits dual therapeutic mechanisms: α-thrombin inhibition and anti-thrombotic activity. The bioactive constituents of Hirudo (leech) are primarily administered through dual delivery pathways: enteral absorption via oral intake and transdermal permeation through topical application. Hirudin, a thrombin-specific polypeptide inhibitor, demonstrates bioavailability contingent upon intestinal mucosal permeability and resistance to proteolytic degradation. Pharmacokinetic investigations reveal that while partial enzymatic cleavage in the gastrointestinal tract converts hirudin into low-molecular-weight peptides, these derivatives maintain substantial bioactivity (Stepensky, 2018). Hepatic and renal systems predominantly mediate hirudin metabolism, yielding amino acid derivatives and bioactive peptide fragments that may modulate endogenous biosynthetic pathways (Yuan et al., 2013). Notably, hirudin’s potent thrombin-inhibiting capacity necessitates cautious clinical application due to dose-dependent hemorrhagic risks and potential toxicological manifestations in coagulopathic patients.

Modern pharmacological studies have demonstrated that blood sugar and lipids in the blood vessels of diabetic patients are prone to deposition. Prolonged deposition can induce inflammation and damage to blood vessels (Lee et al., 2020), as well as result in blood vessel blockage, giving rise to microvascular complications involving the heart, brain, and kidneys (Opazo-Ríos et al., 2020). Hirudin has the ability to regulate blood lipids and exerts a preventive effect against blood vessel blockage induced by diabetes (Cheng et al., 2022). Simultaneously, hirudin can inhibit the inflammatory response via the P38 MAPK/NF - κB pathway and treat kidney injury in diabetic rats (Han et al., 2020). In particular, it can regulate inflammatory factors such as TNF - α, IL - 1, and IL - 6, thereby preventing the occurrence of diabetic nephropathy (Vaidya et al., 2011). In addition to hirudin, the main active components of hirudin analyzed in this network pharmacological study were geniposide, ursolic acid, croomionidine, nadroparin, *etc.*, Geniposide has also been confirmed to have anti-blood sugar effects. *In vitro* studies indicate that geniposide can help lower blood sugar by regulating α-glucosidase. This was confirmed by the analysis conducted by Zhou H et al. using PLC-ESI-QTOF-MS/MS. Additionally, geniposide can regulate the AKT-FOXO1 pathway to inhibit liver glucose production (Yang et al., 2018). Ursolic acid has been shown to reduce postprandial blood glucose levels in patients. Additionally, enzyme kinetics experiments have demonstrated that ursolic acid can inhibit the activities of α-amylase and α-glucosidase, making it an important hypoglycemic component (Wang et al., 2020; Camer et al., 2014).

The results of network pharmacology indicate that the active targets of leeches in diabetes treatment primarily revolve around immune regulation. Studies have found that the main active components of leeches are linked to immune regulatory functions. Hirudin, a natural anticoagulant protein, has demonstrated an anti-thrombotic effect in recent years. This effect is achieved not only through its inhibition of thrombin but also by regulating immune receptors and pathways, which may influence inflammatory responses and immune cell function (Liu et al., 1991). It also functions as a natural antibody, reducing the activity of human T cells (Asgari et al., 2017). Geniposide exhibits a variety of biological activities, including anti-inflammatory, antioxidant, and immunomodulatory effects (Zhou et al., 2019). *In vitro* experiments show that geniposide can enhance the immune response by regulating immune receptors such as STAT and JAK (Gong et al., 2024). It has also been shown to inhibit the expression of TLR4 and MMP9 proteins, thereby influencing the MyD88/NF-κB p65 signaling pathway involved in immune regulation (Liu et al., 2024).

If the active ingredients of leeches and their targets can treat diabetes through immune regulation, what are the specific targets of their effects? To investigate this, we combined the GSE184050 dataset to search for relevant immune cells using single-sample enrichment analysis. Ultimately, five important types of immune cells were identified: activated CD4 cells, activated dendritic cells, central memory CD4 T cells, type 1 T helper cells, and type 2 T helper cells. These five cell types may play an important role in the treatment of diabetes in leeches. To further investigate the correlation between the active components of leeches and immune cells, we conducted WGCNA analysis and identified 26 analysis modules based on the phenotyping of the five immune cells. Among these, the MEdarkturquoise and MElightgreen modules were the most significant, exhibiting the strongest immune correlations in this study. By combining the genes from both modules, a total of 572 module genes were obtained. We intersected the drug genes with the core module genes and identified 122 common targets.

Machine learning can be utilized to identify potential targets by developing predictive models. In this study, to further explore the potential immune targets of leeches in the treatment of diabetes, we constructed a prediction model using 122 intersection targets. This was achieved through LASSO regression, random forest algorithms, SVM-REF, and XGBoost algorithms from machine learning. LASSO regression is an effective analytical method that allows for feature selection in high-dimensional data. It helps prevent overfitting and enhances the predictive performance of the model (Yan et al., 2023). By incorporating L1 regularization terms, LASSO regression compresses the regression coefficients and automatically selects the features that are most relevant to the target variable. To prevent overfitting, we selected the optimal regularization parameter (λ) using 10-fold cross-validation. Ultimately, we identified 16 important variables to construct the model. To further identify genes associated with the disease, we trained the data using a random forest algorithm. This involved constructing multiple decision trees and aggregating the predictions from each tree. Compared to traditional single decision tree models, random forest can more efficiently handle high-dimensional data and prevent overfitting. During the model-building process, we set the number of trees to 56 (ntree = 56), optimizing this parameter through cross-validation to ensure minimal error. Additionally, we calculated the importance of each variable and selected the top 20 variables as disease-related genes. The SVM-REF algorithm combines support vector machine (SVM) and recursive feature elimination (RFE) methods to facilitate effective feature selection (Zhao et al., 2022). In the SVM model, recursive feature elimination optimizes the model by incrementally removing the features that contribute the least to classification, based on the importance of weights or support vectors. This process ultimately retains the features with the greatest classification power. When evaluating the SVM-REF model, we used accuracy and error rate as evaluation metrics. We selected the top 39 variables, which exhibited the highest accuracy and the lowest error rate, to construct the model. The XGBoost algorithm is an effective machine learning model, so we incorporated it into our research. Ultimately, by building this machine learning model, we identified the core targets RGS10, CAPS2, and OPA1.

RGS10 is a member of the RGS protein family, primarily functioning to enhance the GTPase activity of G proteins. This action modulates the signaling pathways associated with G protein-coupled receptors (GPCRs). These pathways are essential for various cellular processes, including metabolism and hormone secretion. Animal studies have shown that RGS10 can help maintain metabolic homeostasis by mitigating inflammatory responses, which in turn reduces insulin resistance (Fang et al., 2019). Additionally, RGS10 is recognized as a significant immune-related target in diabetic retinopathy (Xia et al., 2024). CAPS2 (also known as NLRP3) is an inflammatory body protein that plays a crucial role in the innate immune system. The precise relationship between CAPS2 (NLRP3) and diabetes is not yet fully understood; however, recent investigations suggest that NLRP3 and its associated inflammatory pathways may play a role in the pathogenesis of diabetes (Duan et al., 2020; Jiang et al., 2017). CAPS2 may influence insulin resistance and beta cell dysfunction by regulating the release of inflammasomes and pro-inflammatory cytokines, such as IL-1β (Kelley et al., 2019). Mitochondria are essential energy and metabolic organelles. Diabetes can cause mitochondrial dysfunction, leading to a range of metabolic abnormalities. OPA1 plays a crucial role in regulating mitochondrial fusion. It can mitigate the impairment of mitochondrial fusion caused by diabetes, potentially reducing the development of complications such as diabetic cardiomyopathy (Wang et al., 2023). Research has indicated that OPA1-mediated mitochondrial fusion exerts both regulatory and protective effects against liver and myocardial damage induced by diabetes, primarily through the PKG-STAT3 pathway (Wang et al., 2022; Chang et al., 2023). Existing studies have shown that OPA1 can influence the energy acquisition of dendritic cells by regulating mitochondrial morphology (Virga et al., 2024). We can speculate that a similar effect may also be present in diabetic patients. CAPS2 promotes differentiation of Activated CD4 T cells by initiating Caspase-1-dependent interleukin-1β secretion (Arbore et al., 2016).

To summarize, this study predicts immune targets and regulations using network pharmacology and bioinformatics methods, which could represent novel molecular mechanisms for leeches in treating T2DM. The coordinated multi-target interaction of RGS10, CAPS2, and OPA1 may play a significant role in regulating metabolism, secretion, and mitochondrial function in the treatment of T2DM. However, our study has some limitations. The database analysis is constrained by sample size, which restricts the scope of our research. Additionally, further experimental studies are needed to explore how RGS10, CAPS2, and OPA1 targets dynamically regulate T2DM, particularly how these dynamic changes occur during the early, middle, and late stages of disease development. Current research agrees that leeches possess anticoagulant properties, and anticoagulant treatment proves to be highly beneficial for managing diabetic complications and cardiomyopathy (Bhatt et al., 2020). Therefore, we can anticipate that future studies will further explore the potential of leeches in integrating Chinese and Western medicine through their unique anticoagulant and immune-regulating properties, ultimately benefiting a greater number of diabetic patients.

## Data Availability

The datasets presented in this study can be found in online repositories. The names of the repository/repositories and accession number(s) can be found in the article/Supplementary Material.
